# Supplementary material for: Are carbon emissions trading and green financial instruments synergistic? -Comprehensive quantitative research based on content analysis
Source: PLoS One. 2024 Mar 7;19(3):e0298601. doi: 10.1371/journal.pone.0298601 (PMC10919601; doi:10.1371/journal.pone.0298601)
Supplement: S1 Data — (ZIP) [file pone.0298601.s001.zip › Data/地级市Z_F工作报告.zip!/╔╜╢1⁄2/╢1⁄2╙¬╩╨/└┤╫╘╣1⁄2╓┌║┼í╛┬φ┐╦╩2╛▌═°í┐_2019.docx]

各位代表：

现在，我代表市人民政府向大会报告工作，请予审议，并请市政协委员和其他列席会议的同志提出意见。

一、2018年工作回顾

过去的一年是极不平凡的一年。在中共东营市委的坚强领导下，全市上下以习近平新时代中国特色社会主义思想和党的十九大精神为指导，全面贯彻落实习近平总书记视察山东重要讲话、重要指示批示精神，坚决贯彻党中央、国务院和省委、省政府决策部署，深入贯彻市委六届四次、五次全会精神，迎难而上，砥砺前行，较好地完成了经济社会发展各项目标任务。全年经济增长4.5%；一般公共预算收入244.6亿元，增长5%；城乡居民人均可支配收入分别达到47912元、17485元，增长7%、7.6%。

（一）动能转换迈出新步伐。积极谋划新旧动能转换重大工程，实施和储备优选项目144个，其中18个列入省重点项目和省优选项目。加快工业转型升级，实施市级重点项目29个，威联芳烃等重大项目前期工作扎实推进，国瓷公司入选省高新技术企业创新能力100强，6家中小企业成为省级隐形冠军。积极发展现代高效农业，实施康华海洋牧场、澳亚乳制品加工等过亿元项目5个，新增新型农业经营主体258家。加快发展现代服务业，实施黄河三角洲大数据港、光谷未来城等过亿元项目18个，新增3A级以上景区9家。获批创建国家创新型城市，新增省级以上科研机构40家、高新技术企业55家，新增中国驰名商标1件、山东名牌产品7个、马德里国际商标287件，高新技术产业比重达到34%。

（二）城乡面貌发生新变化。国家方志馆黄河分馆、市档案馆投用，南一路快速路竣工通车，中心城442处小区单位完成雨污分流改造，东城商贸城综合整治、西城管道燃气等工程完工，新建地下综合管廊7.5公里。中心城建设改造湿地公园3个、街头游园和口袋公园19个，广利河森林湿地公园向市民开放。开展城市精细化管理十大专项行动，拆违拆临成效显著。全市棚户区改造开工6208套。编制了乡村振兴战略规划和海洋强市建设行动方案。实施农村人居环境整治，新建改建“四好农村路”240公里，农村无害化卫生厕所建设扎实推进，12个黄河滩区村旧村台改造开工建设，造林绿化9.1万亩。342.7万亩耕地得到有效保护。入选全球首批国际湿地城市，修复自然保护区湿地1.1万亩。重大基础设施建设扎实推进。京沪高铁二通道天津－东营－潍坊段预可研报告通过专家审查。东营港疏港铁路、10万吨级航道建设进展顺利。胜利机场航班达到20个、通航城市12个，中国商飞C919大飞机成功转场东营试飞。油气管网、供电设施等加快建设。

（三）三大攻坚战取得新成效。坚持政监银企联动，一企一策化解资金风险，依法打击恶意逃废金融债务、非法集资等行为，有效维护了金融秩序稳定；坚持防风险与促发展并重，支持引导银行机构优化信贷结构、扩大信贷投放。加强涉油储运等重点行业领域整治，建立危化品运输车辆全过程、一体化、闭环式监管体系，生产安全事故起数、死亡人数实现双下降。加大生态环境污染整治力度，狠抓中央和省环保督察反馈问题整改，实施了一批水气土污染治理项目，省控以上河流稳定达到五类水质，环境空气质量综合指数改善率居全省第1位。强化刑责治污，严格执法监管，严惩环境违法行为。开展打击非法调和销售成品油、违规销售柴油专项行动。脱贫攻坚成果得到巩固提升，对外援建、下派帮扶工作成效明显。

（四）改革开放开创新局面。加大“放管服”改革力度，市级公布“一次办好”事项1460项，完成“3545”改革任务。商事制度改革受到国务院通报表扬，新注册市场主体3.7万户。完成规模企业公司制改制140家，新增挂牌企业37家。深化国企国资改革，实现市属经营性国有资产统一监管。96.4%的村居完成农村集体产权制度改革，县级农村产权交易市场全部投入运营。胜利油田“四供一业”及其他办社会职能移交、共建石油大学胜利学院扎实推进，农高区、综合训练基地加快改革发展，胜利职业学院恢复办学。对内对外开放不断扩大。在建招商引资项目278个。实现外贸进出口1627.9亿元，增长20.2%，总量跃居全省第4位，列全国外贸百强城市第56位。新批外资企业13家，实际使用外资10.6亿元。

（五）民生事业实现新进步。城镇新增就业5.1万人。城市低保标准提高到每人每月600元，农村低保标准提高到每人每年4800元，基础养老金标准提高到每人每月163元。新建改建幼儿园26所、中小学37所。市全民健康信息平台建成投用，组建医联体19家，家庭医生签约服务72.5万人。省级食品安全城市、农产品质量安全市创建成效明显，全国文明城市复审、国家公共文化服务体系示范区创建通过验收。成功举办国际马拉松赛、公路自行车赛。积极应对罕见台风强降雨灾害，灾后重建基本完成。认真落实领导干部公开接访等制度，解决了一批群众反映的突出问题。强力推进扫黑除恶专项斗争，加强社会治安综合治理，创新基层社会治理方式，全力打造“雪亮工程”新名片，群众安全感明显提升。双拥工作深入开展，国防动员、人民防空、民兵预备役建设不断加强。人口计生、慈善救助、工青妇、残疾人、老龄事业加快发展，民族宗教、外事侨务、驻外联络、统计、对台、口岸等工作实现新进步，中央、省驻东营单位作出了重要贡献。

过去一年，我们坚持把党的政治建设摆在首位，把全面从严治党贯穿政府工作全过程。认真执行市人大及其常委会决议决定，自觉把民主政治协商纳入决策程序，办理人大代表建议236件、政协提案250件。全面启动政府机构改革。坚持求真务实，注重调查研究，推进依法行政，履职担当能力不断提高。

各位代表！过去一年的成绩来之不易。这是以习近平同志为核心的党中央坚强领导的结果，是省委、省政府正确领导、关心支持的结果，是市委团结带领全市人民开拓进取、埋头苦干的结果，是市人大、市政协和社会各界监督支持、共同奋斗的结果，是油校军、农高区各方和各位老领导老同志鼎力相助、共襄发展的结果。在此，我代表市人民政府，向全市人民，向各位市人大代表、政协委员和各界人士，向所有为东营发展作出贡献的同志们、朋友们，表示衷心的感谢并致以崇高的敬意！

在肯定成绩的同时，我们也清醒地看到：经济下行压力依然很大，部分企业资金紧张、经营困难；大项目好项目较少，新旧动能转换步伐不快，科技创新能力不强，“四新”经济比重较低，产业结构调整任务艰巨；环境问题仍然突出，安全生产压力巨大；社会事业发展不平衡不充分，民生工作与群众对美好生活的期盼还有差距；部分政府工作人员的专业素养和专业能力不高，工作不认真不扎实，等等。对这些问题，我们将落实针对性措施，认真加以解决。

二、2019年目标任务和总体要求

当前，世界经济形势复杂严峻、变数增多，国内经济运行稳中有变、变中有忧，我市发展压力前所未有。困难虽大，希望更大；挑战虽多，机遇更多。我们的信心来自于市委的坚强领导。市委六届四次全会描绘了宏伟蓝图，确定了“打造山东高质量发展的增长极，黄河入海文化旅游目的地，建设富有活力的现代化湿地城市”的目标定位；刚刚召开的全市经济工作会议，又谋划了一系列战略举措，加强制度设计、制度创新，扎实推进各项重点工作，拉开了今年东营发展的大幕。我们的信心来自于外部环境的积极变化。我国发展仍处于并将长期处于重要战略机遇期，中央、省对民营经济发展高度重视关心，省委、省政府作出加快新旧动能转换、规划建设鲁北高端石化产业基地、开展高质量“双招双引”、打造乡村振兴齐鲁样板、做好经略海洋文章、补齐基础设施短板等重大部署，出台了一系列含金量高的政策措施，必将促进东营的资源优势、基础优势、空间优势加快转化为发展优势。我们的信心来自于全市日益浓厚的发展氛围。干部群众精神振奋、人心思进，广大企业克难转型、求新图强，油地校及农高区精诚团结、同频共振，形成了推动发展的强大合力。只要我们锁定目标、乘势奋进，撸起袖子加油干，加快节奏抓落实，就一定能将美好愿景变成现实，走在山东高质量发展的前列。

2019年是新中国成立70周年，是决胜全面建成小康社会第一个百年奋斗目标的关键之年，是全面落实市委六届四次全会部署的开局之年。做好今年政府工作，要以习近平新时代中国特色社会主义思想为指导，认真贯彻党的十九大和十九届二中、三中全会精神，深入落实习近平总书记视察山东重要讲话、重要指示批示精神，坚持稳中求进工作总基调，坚持新发展理念，坚持推动高质量发展，坚持以供给侧结构性改革为主线，坚持深化市场化改革、扩大高水平开放，以新旧动能转换重大工程为引领，紧紧围绕新时代东营发展的目标定位，全力实施融合、聚焦、开放“三大战略”，突出抓好项目建设、“双招双引”、环境打造、金融动能转换“四项重点工作”和五个“三年行动计划”，继续打好三大攻坚战，统筹推进稳增长、促改革、调结构、惠民生、防风险工作，保持经济运行在合理区间，进一步稳就业、稳金融、稳外贸、稳外资、稳投资、稳预期，促进经济持续健康发展和社会和谐稳定，以优异成绩庆祝中华人民共和国成立70周年。

主要预期目标是：经济增长6%左右，一般公共预算收入增长5%，城乡居民人均可支配收入分别增长7%左右。全面完成省下达的约束性指标。

实现上述目标，必须牢牢把握新时代东营发展目标定位这个根本牵引，一切举措围绕目标定位制定，一切工作围绕目标定位展开，以目标定位牵动全局，以目标定位凝聚力量；必须牢牢把握稳中求进这个工作总基调，坚持“稳”字当头，着力推动更高层次、更可持续的“进”，实现以“进”促“稳”；必须牢牢把握推动高质量发展这个根本要求，全面贯彻新发展理念，更加注重发展先进制造业，更加注重以“四新”促“四化”，更加注重安全环保，加快新旧动能转换；必须牢牢把握改革开放创新这个根本动力，坚持以解放思想为先导，全力突破全局“重点”、发展“难点”、民生“痛点”、市场“堵点”、制度“支点”，不断塑造发展新优势；必须牢牢把握以人民为中心这个根本导向，多办民生实事，多解民生难事，努力让人民群众有更加贴身、更加贴心的获得感。

各位代表！市委六届四次全会提出的“三大战略”，符合全市经济发展趋势和变革方向，符合中央和省关于经济工作的总体思路和要求，是我们加快新旧动能转换、推动高质量发展的重要战略路径，必须随着形势的发展变化，不断赋予新的内涵，坚定不移推进实施。

坚定不移实施融合战略，推进产业融合、产城融合、区域融合。融合发展激发内生动力。从产业融合抓起，积极发展农业“新六产”，集中抓一批专业特色小镇和田园综合体；运用“互联网+”“人工智能+”改造提升传统产业，提高产业智慧化水平，迸发产业发展新活力。产业是城市的支撑，城市是产业的载体，产城融合才能实现精明增长。城区改造、片区开发要以产业为先导，合理配置功能，科学导入现代城市产业，实现城市与产业时序上同步演进、空间上分区布局、功能上产城一体，以产兴城、以城促产、产城融合。区域融合是发展的必然。东营经济技术开发区、东营区要高点定位，以更高标准推动规划建设管理，着力打造精致城市。垦利区要纳入中心城区，切实树立市区理念，推动与主城区相向发展。加快油地校融合，发挥胜利油田、石油大学更大作用，合力促进区域经济社会发展。农高区是东营的重要组成部分，要全面加强规划对接、产业对接、设施对接，着力建设地区创新中心。

坚定不移实施聚焦战略，推进产业聚焦、区域聚焦、体制机制聚焦。握指成拳，切实抓好事关大局、事关长远的工作。聚焦重点产业。制定市“九大”产业发展规划，推动石化、橡胶产业加快整合提升、转型发展，新材料、航空航天产业壮大规模、形成新亮点。聚焦重点区域。东营港经济开发区要落实“港区融合、三位一体”，统筹规划，完善设施，以一类对外开放口岸和鲁北高端石化产业基地核心区的标准推进工作。东营经济技术开发区要启动新一轮发展规划，创新产业、创新体制机制，建设特色专业园区，着力推动产业集群发展、规模扩张和质量提升，担当起全市新旧动能转换主战场、“双招双引”主阵地、产城融合示范区的重任。黄河口生态旅游区要整合周边资源，配套基础设施，完善旅游功能，打造黄河入海文化旅游目的地核心区。金湖银河片区综合开发与北二路及东营河沿线改造，要围绕湿地特色，突出医养健康等现代产业，建设区域价值高地。聚焦体制机制创新。坚持深化改革，全面理顺市级、区级城市管理体制，推进管理重心下移。按照“多予、少取、放活”的原则，实施市以下财政管理体制改革，增强县区财政保障能力，优化市区建设管理事权与财权，加强财政预算绩效管理。进一步向县区、开发区放权，实现“市县同权”，省级以上开发区实现“一枚印章管审批”。加快园区改革，按照“大部制、扁平化”和“企业化管理、市场化运作”的思路，全面实行以“KPI”考核为核心的目标管理和绩效考核办法，激发园区发展活力。继续推动“个转企、小升规、规改股、股上市”，重点培育70个“小升规”企业、100个“规改股”企业。深化国企国资改革，优化资本布局结构，推进县区经营性国有资产统一监管。优化“3+N”“1+N”快速决策、重点项目推进、“双招双引”促进、油地校融合等机制，推动各项重点工作高效落实。

坚定不移实施开放战略，推进思想解放、队伍建设和对内对外全面开放。思想解放是开放的先导。巩固扩大省内对标学习青岛、济南高新区和省外学习杭州、苏州工业园成果，以敢想敢干、敢为人先的精神推动改革开放再出发。不断增强开放意识、开放能力，着力建设“双招双引”、外经贸工作专业队伍，切实提高干部队伍专业素养。按照“法无禁止皆可入”的要求，全面落实市场准入负面清单制度。深入实施“境外百展市场开拓计划”，积极应对国际贸易摩擦，扶持骨干外贸企业创新发展，稳定出口规模。大力发展服务贸易，培育跨境电商、外贸综合服务等外贸新业态。优化口岸营商环境，整体通关时间压缩10%以上。引进德国博世马勒等高质量外资项目，以项目带技术、带理念、带管理、带市场。支持企业开展国际并购、资产重组、合作研发，深度融入“一带一路”建设。继续办好“五大展会”。东营综合保税区要抓住国家促进综保区升级的机遇，打造对外开放的重要窗口。积极承接北京非首都功能疏解和产业转移，深化与港澳台合作，加强与日韩、欧美等国家经贸往来，向海内外投资者敞开大门，构建对外开放新高地。

三、2019年重点工作

完成好全年任务，“四项重点工作”是关键，五个“三年行动计划”是抓手。要按照年度工作安排，落实路线图、时间表、责任人，推动各项重点工作落地见效。

（一）紧抓第一要务，着力促进经济高质量发展。东营正处在爬坡过坎、滚石上山、转折转型的关键时期，必须紧紧抓住发展这个第一要务，认真贯彻落实“巩固、增强、提升、畅通”八字方针，立足制造业强市加快调结构、转动能，推动高质量发展。

加快新旧动能转换。加强项目建设、推进新旧动能转换，是东营发展的当务之急。今年重点实施总投资1024亿元的100个产业项目，强化投资拉动，加快构建现代产业体系。推动特色产业做强做优。坚持“工业为主、企业为尊、集群发展”，实现制造业浴火重生。石化产业，大力推动炼化产能优化整合，加快实施威联芳烃等高端项目，积极争取布局炼化一体化项目，推动石化产业由原材料产品、中间产品向高层次市场终端产品发展。橡胶产业，引导轮胎企业注重品牌、强强联合，打造行业龙头企业，尽快实现转型升级、脱胎换骨。新材料产业，实施国瓷特种陶瓷材料、利华益高端工程塑料等重点项目，规划建设国瓷新材料产业园，积极引导石化、有色金属等企业向新材料领域延伸发展，加快形成千亿级产业集群。石油装备产业，推动科瑞、高原等骨干企业与国企合作重组、与外资企业合资合作，提高创新能力和智能制造水平。有色金属产业，依托方圆等企业，进一步提高冶炼技术水平，延长产业链条，向高强高导的铜合金材料、高性能金属材料发展。支持造纸、纺织、盐化工等产业转型升级。注重工艺优化和节能环保，大力推进新一轮技术改造和设备更新，利用新技术新装备推动“老树发新芽”。深入落实“企业上云”实施方案，着力培育一批上云标杆企业。推动优势产业发展壮大。落实“旅游富民”三年行动计划，把文化旅游产业发展成为富裕东营的支柱产业、幸福东营的惠民产业和宜居东营的生态产业。引进华侨城等旅游龙头企业，按照“政府做线路、企业做项目、合力做市场”的思路，充分利用“河海交汇、新生湿地、野生鸟类”三大世界级旅游资源，集中打造黄河口生态旅游区和沿黄、滨海两条精品线路，打响“黄河入海，我们回家”旅游品牌。充分挖掘红色文化、石油文化、黄河文化、吕剧文化等资源，丰富旅游文化内涵，形成文旅融合发展新优势。规划实施黄河口文旅小镇、黄河文化馆提升、黄河科技馆布展、石油科技馆和耿井休闲体育公园建设等项目，开通湿地观鸟科普游线，依托现代海洋牧场平台等新载体，在观览河海交汇景观上率先突破。加快发展现代高效农业，坚持用工业化的理念推进农业现代化，以发展工业园区的模式建设农业园区，围绕黄河口大米、大闸蟹、海参、奶牛、生猪、肉羊、林果、花卉等产业，培育和引进一批农业龙头企业，加大黄河口名优农产品品牌建设力度，实施智慧农业物联网硅谷、华澳大地牛奶小镇、黄河故道现代农业示范区、黄河生态经济带等重点项目，提升产业发展水平。推动未来产业快速起步。加快培育航空航天产业，完善航空航天产业园规划，依托胜利机场加快中国商飞东营试飞基地建设，积极论证打造南北中转枢纽机场，全力推进空客再交付中心等项目。加快培育生物医药产业，依托化工产业拓展延伸，积极发展生物化工、制药化工、精细化工及其配套产业，实施天东公司依诺肝素钠等重点项目，壮大产业规模，形成集聚效应。加快推进建筑业改革发展，打造“东营建设”品牌。把发展服务业与扩大消费需求结合起来，促进传统服务业转型升级，发展壮大现代物流、电子商务等现代服务业，实施中科创新广场、鲁北铁路物流园等重点项目，加快补齐服务业短板。强化海洋意识，实施海洋强市建设行动方案，重点发展海洋渔业、海洋装备、海洋生物医药等产业，扎实推进海洋重大项目建设，切实保护海洋生态环境，做好经略海洋文章。

大力开展“双招双引”。落实“双招双引”三年行动计划，完善专门招商机构，实施专业精准招商。突出产业招商，围绕省“十强”产业、市“5+2+2”产业体系、“四新”产业，紧紧盯住世界500强企业、国际国内综合实力突出的旗舰型企业、细分领域的隐形冠军企业、高科技领域的独角兽企业，开展敲门式招商，引进一批建链、延链、补链、强链项目，推动中国一汽华东智能网联汽车试验场、广汽新能源汽车等项目尽快落地。重视企业招商，引导企业加强对外合作，引入上下游配套企业。围绕高端石化产业基地建设，既要加强与国企的重组合作，又要抓住国家放开石化产业外资准入、外资企业竞相到我国投资布局的机遇，争取引进资金实力雄厚、技术优势突出的知名外企，实现石化产业持续发展、高效发展。充分发挥开发区、园区重要作用，引进聚集一批高端产业项目，培育优势突出、特色鲜明的“四新”板块和产业集群。坚持产才互动、以用为本，着力引进一批行业领军人才、创新创业团队和专业技能人才。营商环境是“双招双引”的生命线。突出落实“一次办好”，推动“3545”走深走实。进一步优化审批流程，积极推行容缺受理、多评合一等模式，推进“证照分离”、企业登记全程电子化，实施“双随机、一公开”监管。加快“互联网+政务服务”建设，实现政务服务事项数据“全打通、全归集、全共享、全对接”。认真落实减税降费措施，实行政府解决企业难题直通车制度，开展“百名局长服务百家企业”活动，为企业送政策、送优惠、送服务，支持民营企业发展，以与企业家赛跑的精神为企业服务。

注重提升创新能力。以创建国家创新型城市为抓手，牢牢把握创新平台、科技人才两个关键，推进产学研结合、油地校结合、科技金融结合，加快提升科技创新能力。围绕重点产业，扎实推进多方共建、多元投入、混合所有、团队为主的产业技术研究院建设，并探索赋予管理人员、科研人员更多自主权。发挥油地校资源互补优势，共建科研聚集区，聚焦产业需求开展技术研发，推动科技成果就地转化。依托农高区，建设国家现代农业技术创新中心，汇集中科院20余家院所、中国农业大学、山东农业大学等科研力量，建设一流的现代农业科技创新高地、农业高新技术产业孵化基地、现代农业技术辐射中心。东营高新区要充分发挥国家大学科技园的优势，建成科技型中小企业成长的摇篮。加大财政投入和税收减免力度，强化“创新券”等普惠性政策支持，引导企业建立研发准备金制度，支持有条件的企业建设离岸科研机构，加强科技研发，充分发挥企业创新主体作用。我市中小企业数量多、潜力大，要全力支持中小企业创新发展，大力培育瞪羚企业、独角兽企业和行业隐形冠军。支持企业用好科技创新、品牌创新、管理模式创新“三大法宝”，心无旁骛攻主业，不断提升核心竞争力。大力推进质量强市建设。加快国家知识产权强市创建和中国（东营）知识产权保护中心建设，有效发明专利拥有量增加14%以上。着力培育企业家和创新型人才队伍，激发创新创造活力，优化企业家干事创业环境，让重视科技、崇尚创新在全社会蔚然成风。

（二）强化底线思维，着力打好三大攻坚战。聚焦防范化解重大风险、精准脱贫、污染防治硬任务，既补短板，又固底板，为高质量发展创造良好环境。

打好防范化解重大风险攻坚战。以落实金融动能转换三年行动计划为统领，统筹解决金融领域突出问题，促进经济金融深度融合。鼓励银行机构积极争取政策支持，围绕重点产业、重点项目，加大信贷投放力度。支持企业上市挂牌，加快组建新旧动能转换基金群，拓宽企业融资渠道。加强金融风险监测预警，因企施策化解企业风险，确保金融安全形势有效稳控。加快推进政府背景融资担保公司增资扩股，加强与大型担保机构业务合作，探索政银企共担风险模式，化解大型优质企业担保圈风险。加强社会诚信体系建设，积极开展形式多样的银企对接合作活动，深入推进银企互信企业名单制管理工作，为优质诚信企业提供稳定可靠的融资保障。坚决打击恶意逃废金融债务行为，扎实做好防范处置非法集资工作，优化金融生态环境。支持地方法人银行机构稳健发展，加快东营银行上市步伐，鼓励证券、保险行业加快发展。高度重视安全生产，以创建全省安全发展示范城市为契机，持续加强危化品、道路交通、建筑施工、海上作业、公共场所等重点领域整治，加强风险隐患双重预防体系建设，提高应急管理能力，确保安全生产形势持续稳定。

打好精准脱贫攻坚战。深化专项扶贫、行业扶贫、社会扶贫“三位一体”大扶贫格局，巩固“两不愁三保障”成果，完善“两建一推六保障”机制，推动从解决贫困问题向巩固脱贫成效转变，从单点扶贫向连片开发转变，从集中突破向完善长效机制转变。促进贫困人口转移就业，完善保障性扶贫措施，同步解决贫困群众脱贫与解困问题。全力支持黄河滩区建设发展，扎实推进旧村台改造提升。高度关注群众因病致贫、因病返贫，全面小康路上不让一个人掉队。

打好污染防治攻坚战。环境污染已经成为制约东营长远发展、影响东营形象的突出问题。深入贯彻习近平生态文明思想，坚决抓好中央、省环保督察反馈意见和“回头看”移交问题整改，持续治理非法调和销售成品油、危废固废、空气异味、城市扬尘等突出问题。深入推进生态环境保护和“四减四增”三年行动计划，坚决打好8场标志性战役，全面落实河长制湖长制湾长制，加强环渤海排污口排查整治，实施广利河溢洪河流域及城市内水系环境综合治理、危险废物综合处置中心等93个水气土污染治理项目，确保空气优良率达到60%以上，市控以上河流基本达到五类水质。严格落实企业主体责任，健全生态环境损害赔偿、排污许可、环保信用评价等制度。强化环保执法监管，完善有奖举报制度，坚决严厉打击污染物偷排偷放、生态环境破坏、监测数据造假等环境违法违规行为，让东营的天更蓝、水更清、空气更洁净。

（三）注重精明增长，着力提升城市功能品质。围绕内涵提升、品质建设，努力打造市民满意的城市、人才向往的城市、游客点赞的城市、投资者称道的城市。

突出规划引领。坚持产城融合、多规合一，搞好新一轮城市总体规划修编，优化城市空间布局，形成“一主一副一次三组团、多地区中心”的格局。开展水资源配置利用、综合交通体系建设等专项规划，科学编制中心城水系连通、水循环利用和水生态治理方案，完成湿地城市总体设计。规划控制高铁片区，条件成熟时开发建设。促进县城和小城镇品质提升、创新发展，培育一批各具特色的魅力城镇。支持河口区与东营港区协同发展，支持广饶县进位争先，推动利津县跨越发展。

突出建设管理。坚持精建细管、完善功能，以迎接国家生态园林城市验收为契机，提高城市建设管理规范化水平，着力打造生态品牌。加强中心城区房地产用地管理，健全土地熟化机制，规范土地供应。打通华山路等8条断头路，建设一批小绿地、小公园。实施东八路生态环境综合整治工程，建设城市湿地景观长廊。坚持用生态的办法解决生态问题，年内贯通登州路、菏泽路等5条中心城水系。实施以水工程为主的自然保护区生态修复和保护工程，修复湿地3万亩。坚持宜湿则湿、宜林则林，新增造林7.5万亩。深入推进胜利油田办社会职能移交工作，让广大油田职工享受到更优质的公共服务。

突出基础支撑。重点补齐交通短板，加快京沪高铁二通道天津-东营-潍坊段前期工作，完成高铁站选址，力争年内开工建设黄河高铁大桥；建成东青高速何官互通立交。东营港疏港铁路年底达到试运营条件，黄大铁路（东营段）年内建成。长深高速广饶至高青段建成通车，开工建设兴河大道，实施桩埕路改造，济南-高青高速公路力争达到开工条件，加快东青高速公路扩容改造前期工作。完成东营港南防波堤、10万吨级航道工程主体；完善港口建设规划，充分发挥渤海湾港口集团作用，加快推进10万吨级液体散货泊位建设，支持广利港发展。加快原油成品油管网建设。加密胜利机场至上海、广州、深圳等城市的航线，尽快实现东营至一线城市当天往返。

（四）围绕城乡融合，着力实施乡村振兴战略。按照“产业兴旺、生态宜居、乡风文明、治理有效、生活富裕”的总要求，聚焦“五大振兴”，扎实推进农村人居环境整治三年行动，打造城市人的休闲地、农民就业地、社会资本投资地。

聚力建设特色示范片区。坚持市级统筹、县区主体、乡村实施、社会参与，在要素配置和公共服务等方面集中用力，整合涉农资金，支持每个县区建设1-2个各具特色的乡村振兴示范片区。挖掘民俗历史文化，编制特色鲜明的村庄规划，建成一批美丽村居。聚力改善农村人居环境。加强县乡公路建设改造，推进城乡交通运输一体化。巩固燃气村村通成果，鼓励使用地热等安全高效的清洁能源。完善农村厕所后续管养机制，建设188处农村生活污水处理设施。继续推进黄河南展区新型农村社区建设。实施124个农田水利基本建设项目，推进田水路林综合治理。聚力推进农村综合改革。大力培育新型农业经营主体，新增农民合作社70家、家庭农场100家。深入推进农村集体产权制度改革，完善农村产权交易体系，推进资源变资本、资金变股金、农民变股民，促进农村发展、农民增收。推进涉农金融创新，提高农村金融服务水平。扎实开展新型职业农民认定和职称评定试点。健全城乡融合发展体制机制和政策体系，促进资本、技术、人才、管理等先进要素流向农业农村，让广大乡村活力迸发。

（五）坚持以人为本，着力发展社会民生事业。民之所望，政之所向。必须突出民生优先，扎实做好保障和改善民生工作，使人民群众有更多、更直接、更实在的获得感、幸福感、安全感。

强化就业保障。把稳就业摆在突出位置，落实更加积极的就业创业政策，完善全市统一的服务平台，重点解决好高校毕业生、农民工、退役军人等群体就业。切实做好农民工工资保障工作。深入实施全民参保计划。适度提高居民医保财政补助标准，完善医保支付制度，扩大居民长期照护保险试点范围。加强城乡低保和特困群体救助工作，健全失独家庭和农村“三留守”人员关爱服务体系，努力做到应保尽保。

发展社会事业。美好生活从美好教育抓起，优化学前教育、义务教育布局，新建改建城乡中小学26所。推动高中阶段教育多样化特色化发展。支持高等教育、职业教育加快改革发展，重视企业员工继续教育，深化产教融合、校企合作，为产业发展培育人才。建立健全扶持政策，鼓励社会力量兴办教育。加强教师队伍建设，不断提高教师地位待遇，全面提升教师队伍素质能力。积极推进健康东营建设，完善医药卫生体制，有序实施分级诊疗制度，深化医联体建设，全面推行县乡医疗卫生一体化；深化公立医院改革，健全运行机制，完善薪酬制度，提升医疗服务水平；组建市中医院、市传染病医院、市精神卫生中心，开工建设市妇幼保健中心综合楼；深入开展爱国卫生运动，通过国家卫生城市复审，积极创建全省首批健康城市。弘扬优秀传统文化，大力发展文化事业。统筹发展群众体育、竞技体育和体育产业，办好国际马拉松赛、公路自行车赛和市第十一届运动会，创建国家全民运动健身模范市。深入实施养老服务提升行动，促进医养结合。支持军队建设改革，加强国防后备力量建设，深化双拥共建，推进军民融合发展。加强退役军人管理和服务保障制度体系建设，切实维护军人军属合法权益。做好民族宗教工作。积极发展老龄、残疾人、慈善和社会福利事业，让城市更有“温度”。

创新社会治理。落实“夯实基层基础”三年行动计划，加快整合综治信访、安监环保、城市管理、食药监管等各类网格，配齐配强专职化网格员队伍，构建“多网合一、一网运行”的网格化社会治理体系，强化科技和投入保障，提高基层社会治理能力和水平。深入开展“七五”普法，完善公共法律服务体系，着力发挥人民调解作用，预防和化解矛盾纠纷。加强和改进新形势下的信访工作，用心用情解决群众合理诉求。加强“平安东营”建设，深化“雪亮工程”应用，积极创建全国禁毒示范城市，纵深推进扫黑除恶专项斗争，让一切黑恶势力无处遁形。

各位代表！人民对美好生活的向往，就是我们的奋斗目标。在广泛征求群众意见建议的基础上，今年重点办好13件民生实事，总投资19.1亿元。(1)方便市民便捷出行，优化中心城区30个拥堵路口，建设智能信号系统。(2)整治中心城内涝，改造46处积水点。(3)贯通城区部分道路。(4)新建改建城乡幼儿园11所。(5)对部分中小学老旧校舍进行改造。(6)建设改造“四好农村路”277公里。(7)新建15座电动汽车充电站。(8) 实施民生综合保险。 (9) 建设15处城市社区养老服务中心。 (10)加强乡镇街道和社区公共健身设施建设。(11)提升公共卫生服务能力，开展严重精神障碍患者诊治救助，加强预防接种门诊标准化建设。(12)增强基层公共文化服务效能，新建50个数字文化广场，扶持发展20个社会文艺团体（庄户剧团）。(13)开通东营到济青高铁青州北站、临淄北站等专车，让群众方便地乘坐高铁。民生工作无小事，我们一定从群众身边的事情做起，一件接着一件办，一年接着一年干，努力让全市人民的生活更幸福更美好！

四、加强政府自身建设

人民满意是政府工作的最高标准。我们将牢记职责使命，不断提升履职能力和水平，以拼搏奋进、砥砺奋斗的扎实业绩，回报全市人民的信任和重托。

要旗帜鲜明讲政治。深入学习贯彻习近平新时代中国特色社会主义思想，树牢“四个意识”，坚定“四个自信”，坚决做到“两个维护”，在思想上政治上行动上同以习近平同志为核心的党中央保持高度一致。扎实开展“不忘初心、牢记使命”主题教育。不折不扣贯彻党中央决策部署，抓好省委、省政府和市委各项工作落实。始终把人民放在心中最高位置，做到群众有需求、政府有行动。

要依法行政提效能。当前，政府工作面临的任务重、矛盾多，我们要立说立行、雷厉风行，以坐不住、等不起的紧迫感责任感，大力推动工作提速提效，打造精简高效的政务环境。健全规范决策咨询、专家论证等程序，提高决策科学化、民主化、法治化水平。完成机构改革任务，按照新体制高效运行。深化“放管服”改革，推进综合执法和政务公开，对违法者“利剑高悬”，对守法者“无事不扰”。加强政府立法、行政执法监督和行政复议工作，完善行政机关负责人出庭应诉机制。自觉接受人大及其常委会法律监督、政协民主监督和社会监督，高质量办理人大代表建议和政协提案。

要改进作风抓落实。把今年作为“工作落实年”，坚持实在、实干、实绩，改进考核方式方法，紧盯不敬畏、不在乎、喊口号、装样子四类问题，严厉整治形式主义、官僚主义。针对不落实问题，深化“啄木鸟”行动，广泛深入开展舆论监督。全体政府工作人员要勇于直面问题，敢于攻坚克难，坚定信心、苦干实干，始终保持良好精神状态。把认真办事挺在前面，对重点工作实行台账式管理、项目化推进，以严实作风抓落实。要牢固树立过紧日子的思想，从严控制一般性支出，市级对业务类项目支出统一压减10%。深入推进学习型政府建设，提高战略思维能力、研究谋划能力、融合合作能力，建设高素质公务员队伍。

要正风肃纪树形象。坚定扛起全面从严治党政治责任，扎实履行“一岗双责”。严格落实中央八项规定及实施细则精神，聚焦公共资金、国有资产、工程建设、民生保障等重点领域，扎牢织密制度笼子，零容忍惩治腐败。加强审计监督，强化审计结果运用。坚持严管与厚爱相结合，激励广大干部担当作为，营造风清气正的政治生态，汇聚干事创业的强大正能量。

各位代表！新一轮发展风鹏正举，新一年工作重任在肩。让我们更加紧密地团结在以习近平同志为核心的党中央周围，以习近平新时代中国特色社会主义思想为指导，在省委、省政府和市委的坚强领导下，解放思想，改革创新，真抓实干，奋力争先，为打造山东高质量发展的增长极，黄河入海文化旅游目的地，建设富有活力的现代化湿地城市不懈努力，以更加优异的成绩迎接中华人民共和国成立70周年！
